# Dietary N-carbamylglutamate supplementation improves laying performance, lipid metabolism, and antioxidant capacity in Huoyan geese: insights from liver transcriptomics

**DOI:** 10.1016/j.psj.2026.107981

**Published:** 2026-10-17

**Authors:** Xiaobing Peng, Xuan Zhao, Yizeng Lin, Meng Sun, Di Han, Changjiang Li, Ying Wang, Chunchi Yan, Wei Ma, Chunqiang Wang

**Affiliations:** a Department of Animal Science, College of Agriculture, Jinzhou Medical University, Jinzhou City, Liaoning Province, 121000, China; b Liaoning Provincial Key Laboratory of Animal Product Quality and Safety, Jinzhou Medical University, Jinzhou City, Liaoning Province, 121000, China; c Liaoning Provincial Rural and Agricultural Development Service Center, Shenyang City, Liaoning Province, 110000, China; d Liaoning Provincial Modern Agricultural Production Base Construction Engineering Center, Liaoyang City, Liaoning Province, 111000, China

**Keywords:** N-carbamylglutamate, Huoyan goose, Laying performance, Lipid metabolism, Transcriptome

## Abstract

This study investigated the effects of dietary N-carbamylglutamate (NCG) supplementation on laying performance, egg quality, lipid metabolism, and antioxidant capacity in Huoyan geese, and explored the underlying molecular mechanisms through liver transcriptomics. 240 female Huoyan geese of similar body weight and age were randomly assigned to four groups: a control group fed a basal diet and three groups fed the basal diet supplemented with 0.06%, 0.08%, or 0.12% NCG. Each group comprised six replicates of 10 geese. The trial lasted 127 d (7-d adaptation). At the end, laying performance and egg quality were assessed, and serum and liver samples were collected for lipid metabolism and antioxidant biomarker analyses. Liver transcriptomes of the control and N1 groups were sequenced. The 0.06% NCG (N1) group exhibited significantly higher laying rate, average egg weight, eggshell strength, eggshell thickness, yolk weight, and serum high-density lipoprotein cholesterol (HDL-C) levels than the control (*P* < 0.05). Serum total cholesterol (T-CHO), triglyceride (TG), and liver T-CHO, TG, and low-density lipoprotein cholesterol (LDL-C) were significantly decreased in the N1 group (*P* < 0.05). Superoxide dismutase (SOD) activity was significantly enhanced in serum and liver (*P* < 0.05). The response was not monotonic: 0.06% NCG produced the greatest improvements, whereas 0.12% did not differ significantly from the control for most variables. Transcriptomic analysis identified 1,041 differentially expressed genes (DEGs) between groups: 137 upregulated and 904 downregulated in N1. After correction for multiple testing, fructose and mannose metabolism was the only significantly enriched KEGG pathway, and enriched Gene Ontology terms were dominated by ribosome- and mitochondrial respiratory chain-related functions. Gene set enrichment analysis (GSEA) further showed that the ribosome gene set was significantly suppressed in the NCG group, whereas no gene set was significantly upregulated. In conclusion, dietary supplementation with 0.06% NCG improved laying performance, egg quality, lipid metabolism, and antioxidant capacity in Huoyan geese, accompanied by hepatic transcriptomic changes associated with lipid and energy metabolism, antioxidant defense, and mitochondrial function.

## Introduction

The Huoyan goose is a prized indigenous Chinese goose breed valued for its high egg production and strong adaptability, occupying an important position in large-scale poultry production (Romanov, 1999; Yu et al., 2023). However, intensive production systems have increasingly been associated with declining laying rates, deteriorating egg quality, disordered lipid metabolism, and oxidative stress, which collectively constrain the healthy development of the goose industry and reduce farming profitability (Kozák, 2021). Consequently, identifying safe and effective feed additives capable of simultaneously improving laying performance, metabolic health, and antioxidant capacity has become a research priority in poultry nutrition (Yan et al., 2025).

N-carbamylglutamate (NCG) is a structural analog of N-acetylglutamate that activates Δ1-pyrroline-5-carboxylate synthetase (P5CS) and carbamoyl phosphate synthetase I (CPS-I), thereby promoting the synthesis of citrulline from glutamine or proline and ultimately enhancing endogenous arginine synthesis (Frank et al., 2007; Huang et al., 2019). As an activator of endogenous arginine synthesis, NCG offers a practical advantage over arginine supplementation: it is effective at a much lower inclusion level and is therefore a cost-effective alternative in animal production (Wu et al., 2009). Arginine serves as a precursor for nitric oxide (NO), polyamines, and other bioactive molecules involved in vasodilation, immune responses, and cell proliferation (Wu et al., 2009; Jobgen et al., 2006). Given that NO is a key regulatory molecule in lipid metabolism, NCG may modulate systemic lipid metabolism through the arginine-NO pathway. Accumulating evidence has demonstrated that dietary NCG supplementation effectively improves growth performance, meat and egg quality, intestinal health, immune function, and antioxidant capacity in poultry (Hu et al., 2019; Yuan-Hao et al., 2024; Ma et al., 2023a,b; Liu et al., 2024; Peng et al., 2025). Nevertheless, studies on NCG application in laying geese remain scarce, and the functional roles and molecular mechanisms of NCG in regulating laying performance, lipid metabolism, and antioxidant capacity—particularly in the Huoyan goose—have not been systematically elucidated.

The liver is the central organ for lipid metabolism and antioxidant defense in poultry. Unlike mammals, the avian liver synthesizes the vast majority of fatty acids, which not only provide energy but also support systemic cell membrane synthesis (Emami et al., 2020). Hepatic lipid metabolism in poultry is also regulated through the gut-liver axis, in which microbial metabolites and bile acids modulate hepatic lipid handling and contribute to the development of fatty liver haemorrhagic syndrome (Zhang et al., 2026; Guo et al., 2026). Liu et al.(2024) reported, through liver transcriptomic analysis of laying hens, that NCG supplementation upregulated fatty acid oxidation-related genes such as *CPT1A* and downregulated fatty acid synthesis-related genes such as *FASN*, suggesting that NCG modulates hepatic lipid metabolism via the AMPK and PPAR signaling pathways. Additionally, NCG treatment has been shown to significantly elevate NO levels and nitric oxide synthase (NOS) activity in the plasma, liver, and intestine of laying hens (Ma et al., 2021), suggesting that elevated NO—a key signaling molecule and antioxidant—may contribute to alleviating oxidative stress and improving overall health. In broiler chickens, in ovo NCG injection improved growth performance and meat quality, potentially mediated by elevated thyroid hormone and testosterone levels (Yuan-Hao et al., 2024). These findings provide important clues regarding the molecular mechanisms underlying NCG-mediated metabolic regulation in poultry; however, they predominantly focus on chickens, and systematic investigations in waterfowl species such as geese remain limited.

Based on the above evidence, the present study was designed to systematically evaluate the effects of graded dietary NCG supplementation (0.06%, 0.08%, and 0.12%) on laying performance, egg quality, serum and liver lipid profiles, and antioxidant capacity in Huoyan geese during the laying period. Furthermore, liver transcriptome sequencing (RNA-seq) was performed to compare the control and optimal supplementation groups, aiming to identify differentially expressed genes (DEGs) and their enriched metabolic and signaling pathways, thereby elucidating the molecular mechanisms through which NCG improves laying performance and metabolic health. The findings are expected to provide a theoretical basis and technical support for the scientific application of NCG in waterfowl production.

The novelty of this study lies in the hypotheses tested here, none of which has been examined in waterfowl: first, that graded dietary supplementation with NCG improves laying performance and egg quality in Huoyan geese; second, that these improvements are accompanied by measurable changes in lipid metabolism and antioxidant capacity; and third, that the response is not monotonic, so higher supplementation levels do not necessarily produce larger effects. To our knowledge this is the first study to combine production, egg-quality and biochemical responses with a hepatic transcriptome analysis in laying Huoyan geese, which provides a candidate supplementation level for waterfowl production and a set of testable mechanistic hypotheses.

## Materials and methods

### Experimental design, birds, and diets

A total of 240 female Huoyan geese (250 d of age; laying rate 19.0 ± 1.0%) were randomly allocated to four treatment groups: a control group (C) fed a basal diet, and three experimental groups (N1, N2, and N3) fed the basal diet supplemented with 0.06%, 0.08% or 0.12% NCG, respectively. Each treatment group consisted of six replicates, with 10 geese per replicate. The experimental period lasted 127 d, preceded by a 7-d adaptation period. The basal diet (Table 1) was a maize–soybean meal diet formulated for laying geese and was provided in pelleted form; it contained corn 60.00%, soybean meal 18.80%, corn germ meal 3.90%, corn germ cake 3.00%, wheat bran 4.80%, limestone 7.00%, sodium chloride 0.30%, dicalcium phosphate 1.20% and 1.00% of a pelleted premix (Supplementary Table S3). Crude protein, calcium and total phosphorus were determined by chemical analysis (16.57%, 3.13% and 0.649%, respectively) and the remaining nutrient values were calculated from the feed specification. NCG (purity ≥ 99%; Jiangxi Yuanchang Industrial Co., Ltd., Jiangxi, China) was incorporated in the pelleted premix at 60, 80 and 120 g/kg premix, so that the complete diets contained 0.06%, 0.08% and 0.12% NCG, respectively; the amount of carrier in the premix was adjusted so that the four diets were identical in all other respects and were therefore effectively isonitrogenous and isocaloric. The supplementation levels were selected from previously reported effective doses in poultry: 0.08% in laying hens (Ma et al., 2023a; Liu et al., 2024), 0.08%–0.16% in Dagu chickens (Zhang et al., 2021) and approximately 0.10% in late-laying Huainan partridge chickens (Hong et al., 2025). The geese were kept indoors on raised wire-net flooring; each replicate pen housed 10 geese and had an area of approximately 10 m² (4.0 m × 2.5 m). Throughout the trial the geese were kept under natural lighting, at natural (ambient) temperature and with natural ventilation, and they had free access to feed and water. The experimental protocol was approved by the Animal Ethics Committee of Jinzhou Medical University (approval number: JZMULL2026131).

**Table 1:** Ingredient composition and nutrient levels of the basal diet (as fed).

**Ingredient**	**Content (%)**	**Nutrient level**	**Value**
Corn	60.00	ME (MJ/kg)	11.94
Soybean meal	18.80	CP (%)	16.57
Corn germ meal	3.90	Ca (%)	3.13
Corn germ cake	3.00	TP (%)	0.649
Wheat bran	4.80	NaCl (%)	0.344
Limestone	7.00	Lysine (%)	0.721
Sodium chloride	0.30	Methionine (%)	0.357
Dicalcium phosphate	1.20	Methionine + cystine (%)	0.485
Pelleted premix¹	1.00	Arginine (%)	0.926
Total	100.00		

Note: ¹The diet contained 1% of a pelleted premix, the composition of which is given in Supplementary Table S3. Crude protein, calcium and total phosphorus were determined by chemical analysis and NaCl was measured directly; the remaining nutrient values are calculated. ME = metabolisable energy; CP = crude protein; TP = total phosphorus..

### Laying performance and egg quality measurement

Laying performance was evaluated based on laying rate, average egg weight (AEW), average daily feed intake (ADFI), and feed-to-egg ratio (F/E). Eggs were collected daily, and the number of eggs and total egg weight per replicate were recorded. ADFI was calculated as the difference between feed offered and feed refused, divided by the number of geese and the number of days. F/E was calculated as total feed intake divided by total egg weight (g:g).

Egg quality was assessed at the end of the trial. Thirty eggs per treatment (five per replicate) were collected and analyzed within 24 h of oviposition. Egg shape index was calculated as egg width divided by egg length, measured using a vernier caliper. Eggshell strength was determined using an eggshell strength tester. Eggshell thickness was measured at the equatorial region (with shell membranes removed) using a micrometer. Yolk and albumen were separated and weighed individually. Albumen height and Haugh units were determined using an egg quality analyzer. Yolk color was assessed using a Roche color fan.

### Sample collection

At the end of the experimental period, six geese per treatment (one per replicate) were randomly selected for sample collection. Blood samples were collected from the wing vein and centrifuged at 4,000 × g for 10 min at 4°C to obtain serum. Serum aliquots were stored at −80°C for subsequent analyses. After weighing, geese were euthanized by intravenous injection of Euthasol (1 mL per bird; Virbac, Carros, France), and the liver was immediately dissected, snap-frozen in liquid nitrogen, and stored at −80°C for subsequent analyses.

### Serum and liver lipid metabolism and antioxidant biomarker assays

Serum and liver lipid metabolism biomarkers, including total cholesterol (T-CHO), triglyceride (TG), low-density lipoprotein cholesterol (LDL-C), and high-density lipoprotein cholesterol (HDL-C), and antioxidant biomarkers, including superoxide dismutase (SOD), glutathione peroxidase (GSH-Px), and malondialdehyde (MDA), were measured with enzymatic colorimetric kits for the lipid indices (T-CHO, A111-1-1; TG, A110-1-1; LDL-C, A113-1-1; HDL-C, A112-1-1) and with colorimetric kits for the antioxidant indices (SOD, A001-3-2; GSH-Px, A005-1-2; MDA, A003-1-2), all from Nanjing Jiancheng Bioengineering Institute (Nanjing, China), according to the manufacturer's instructions.

### Liver transcriptome analysis

Liver tissue from all six geese of each group was used for library preparation; two libraries per group were lost during pre-sequencing quality control, and the eight libraries that passed quality control (four per group) were sequenced. Total RNA was extracted from liver tissue using TRIzol reagent (Invitrogen, Carlsbad, CA). RNA integrity and purity were assessed by agarose gel electrophoresis and a NanoDrop 2000 spectrophotometer (Thermo Fisher Scientific, Waltham, MA). RNA concentration was quantified using a Qubit 2.0 fluorometer (Thermo Fisher Scientific), and RNA integrity was further evaluated using an Agilent 2100 Bioanalyzer (Agilent Technologies, Santa Clara, CA). Sequencing libraries were constructed using the NEBNext Ultra RNA Library Prep Kit for Illumina (New England Biolabs, Ipswich, MA) according to the manufacturer’s instructions. Library quality was assessed with an Agilent 2100 Bioanalyzer, and qualified libraries were sequenced on an Illumina platform (Illumina, San Diego, CA) to generate paired-end reads.

Raw reads were subjected to quality control, during which adaptor sequences, poly-N reads, and low-quality reads were removed to obtain clean reads. Sequencing quality was evaluated by calculating Q20, Q30, and GC content. Clean reads were aligned to the goose reference genome (GCF_002166845.1_GooseV1.0; https://ftp.ncbi.nlm.nih.gov/genomes/all/GCF/002/166/845/GCF_002166845.1_GooseV1.0/) using HISAT2 (v2.1.0). Read counts were obtained using featureCounts (v1.6.0), and gene expression levels were normalized to fragments per kilobase of exon per million mapped reads (FPKM) using StringTie (v1.3.5). Differential expression analysis between groups was performed using the DESeq2 R package (v1.26.0) based on read count data, with genes meeting the criteria of |log2 fold change| > 1 and adjusted *P* < 0.05 considered differentially expressed. Functional annotation and pathway enrichment analysis were conducted using the Kyoto Encyclopedia of Genes and Genomes (KEGG) database (http://www.genome.jp/kegg/). Statistical enrichment of DEGs in KEGG pathways was evaluated using the clusterProfiler R package (v3.14.0). Gene set enrichment analysis (GSEA) was performed to identify coordinated pathway-level changes across the full transcriptomic profile.

### Quantitative real-time PCR validation

To validate the RNA-seq results, fourteen DEGs were selected for qRT-PCR validation. *NDUFV1, COX5B* and *SDHC* represented oxidative phosphorylation, *TKFC* represented fructose and mannose metabolism, *KEAP1* represented mitophagy and the oxidative stress response, *GSS* represented glutathione metabolism, *NOS3* represented the arginine-NO pathway, *PRDX6* represented antioxidant defence, *APOA2* represented hepatic lipid transport and lipoprotein metabolism, *RPS18* represented ribosome biogenesis, and *EIF4B, CREB3L3, CFAP69* and S*LC6A9* were randomly selected from the DEG set. Total RNA was extracted from liver tissue samples, and cDNA was synthesized using a PrimeScript RT Reagent Kit (Takara Bio, Dalian, China). Real-time PCR was performed on an ABI 7500 Fast Real-Time PCR System (Applied Biosystems, Foster City, CA) using SYBR Premix Ex Taq II (Takara Bio). Optimized cycling conditions were as follows: initial denaturation at 94°C for 5 min, followed by 45 cycles of 94°C for 15 s and 55°C for 15 s (annealing/extension). Each sample was assayed in triplicate to ensure reproducibility. The relative expression levels of target genes were calculated using the 2^−ΔΔCt method (Schmittgen and Livak, 2008), with β-actin as the internal reference gene. Primers used for amplification (Supplementary Table S1) were specifically designed based on sequences available in the GenBank database.

### Statistical analysis

All data were analyzed using SPSS 26.0 (IBM Corp., Armonk, NY). Laying performance data were analysed using the replicate pen as the experimental unit (n = 6 pens per treatment) and the serum and liver biomarkers using the individual goose as the experimental unit (n = 6 geese per treatment, one goose per replicate pen). Egg quality traits were measured on five eggs per replicate pen. Data are presented as means ± SD. Group differences were assessed by one-way ANOVA followed by Duncan’s multiple range test. Differences were considered statistically significant at *P* < 0.05.

## Results

### Laying performance and egg quality

The effects of dietary NCG supplementation on laying performance are presented in Table 2. The N1 group exhibited a significantly higher laying rate compared to the control group (*P* < 0.05). The laying rate in the N2 group was also significantly higher than that in the control group (P < 0.05), whereas the difference between the N3 and control groups was not significant. Average egg weight in all NCG-supplemented groups was significantly higher than that in the control group (*P* < 0.05), with the highest value observed in the N1 group. No significant differences were detected in ADFI or F/E among the four groups (*P* > 0.05).

**Table 2:** Effects of dietary N-carbamylglutamate supplementation on laying performance of Huoyan geese¹.

**Item²**	**C**	**N1**	**N2**	**N3**	**P-value**
Laying rate (%)	40.51±6.53^c^	45.99±6.51^a^	44.24±6.99^ab^	42.07±7.61^bc^	0.032
AEW (g)	126.02±2.26^c^	130.62±1.07^a^	128.18±1.29^b^	128.37±2.13^b^	0.008
ADFI (kg/d)	0.25±0.06	0.23±0.07	0.23±0.07	0.24±0.09	0.754
F/E (g:g)	5.53±0.29	5.31±0.44	5.51±0.45	5.69±0.23	0.481

a–c Means within a row lacking a common superscript differ (*P* < 0.05).

¹Data are presented as means ± SD (n = 6 replicates per treatment, 10 geese per replicate). C = basal diet; N1 = basal diet + 0.06% NCG; N2 = basal diet + 0.08% NCG; N3 = basal diet + 0.12% NCG. NCG = N-carbamylglutamate.

²AEW = average egg weight; ADFI = average daily feed intake; F/E = feed-to-egg ratio.

Egg quality parameters are presented in Table 3. No significant differences were observed in egg shape index or yolk color among the four groups (*P* > 0.05). Eggshell strength in all NCG-supplemented groups was significantly higher than that in the control group (*P* < 0.05), with the N1 group showing the greatest increase (45.3%). Eggshell thickness and eggshell weight in the N1 group were also significantly higher than those in the control group (*P* < 0.05). Yolk weight in the N1 and N2 groups was significantly greater than that in the control group (*P* < 0.05). No significant differences were detected in albumen height or Haugh units among the groups (*P* > 0.05).

**Table 3:** Effects of dietary N-carbamylglutamate supplementation on egg quality of Huoyan geese¹.

**Item²**	**C**	**N1**	**N2**	**N3**	**P-value**
Egg shape index	0.68±0.02	0.67±0.05	0.67±0.01	0.68±0.02	0.823
Eggshell strength (N/cm²)	120.92±15.21^c^	175.72±9.63^a^	162.1±4.54^b^	151.66±6.2^b^	<0.001
Eggshell thickness (μm)	40.87±5.07^c^	59.46±3.22^a^	55.26±1.51^ab^	52.11±2.06^b^	<0.001
Eggshell weight (g)	12.68±0.93^c^	17.12±1.26^a^	14.9±0.87^b^	13.12±1.65^c^	<0.001
Albumen height (mm)	6.94±2.63	9.16±1.74	8.54±1.02	7.86±2.51	0.312
Haugh unit	72.62±10.46	79.84±12.68	78.22±7.98	68.12±19.75	0.427
Yolk weight (g)	36.48±2.81^c^	47.74±2.4^a^	44.72±5.83^ab^	39.52±4.7^bc^	0.006
Yolk color	3.4±0.55	3.4±0.89	3.6±0.55	3.4±0.89	0.895
Relative eggshell weight (%)	10.57±0.42^b^	13.39±1.22^a^	12.48±1.32^a^	10.44±1.52^b^	0.003
Relative yolk weight (%)	30.43±1.96^b^	37.27±1.04^a^	36.80±5.62^a^	31.32±3.16^b^	0.009

a–c Means within a row lacking a common superscript differ (*P* < 0.05).

¹Data are presented as means ± SD (n = 30 eggs per treatment, 5 eggs per replicate). C = basal diet; N1 = basal diet + 0.06% NCG; N2 = basal diet + 0.08% NCG; N3 = basal diet + 0.12% NCG. NCG = N-carbamylglutamate.

²Egg shape index = egg width / egg length.

Relative eggshell weight and relative yolk weight were significantly higher in the N1 and N2 groups than in the control group (*P* < 0.05; Table 3).

### Serum and liver lipid metabolism biomarkers

Serum and liver lipid metabolism biomarkers are presented in Table 4. Serum T-CHO concentration in the N1 group was significantly lower than that in the control group (*P* < 0.05). Serum TG concentrations in the N1 and N2 groups were significantly lower than that in the control group (*P* < 0.05). Serum HDL-C concentration in the N1 group was significantly higher than that in the control group (*P* < 0.05). No significant differences were detected in serum LDL-C among the four groups (*P* > 0.05). Liver T-CHO and LDL-C concentrations in the N1 and N2 groups were significantly lower than those in the control group (*P* < 0.05). Liver TG concentration in the N1 group was significantly lower than that in the control group (*P* < 0.05). Liver HDL-C concentrations in the N1 and N2 groups were significantly higher than that in the control group (*P* < 0.05). No significant differences were observed between the N3 and control groups for most of the measured lipid biomarkers (P > 0.05); serum and liver HDL-C were the exception, both being significantly higher in the N3 group than in the control group (P < 0.05).

**Table 4:** Effects of dietary N-carbamylglutamate supplementation on serum and liver lipid metabolism biomarkers of Huoyan geese¹.

**Tissue**	**Item²**	**C**	**N1**	**N2**	**N3**	**P-value**
Serum	T-CHO (mmol/L)	19.99±1.07^a^	17.77±1.23^b^	18.75±1.34^ab^	19.72±0.82^a^	0.018
	TG (mmol/L)	2.94±0.36^a^	2.1±0.51^b^	2.33±0.34^b^	2.88±0.29^a^	0.009
	LDL-C (mmol/L)	1.59±0.05	1.44±0.14	1.42±0.16	1.5±0.17	0.235
	HDL-C (mmol/L)	2.62±0.21^c^	3.51±0.22^a^	3.22±0.13^b^	3.22±0.16^b^	<0.001
Liver	T-CHO (μmol/g)	10.28±0.91^a^	6.99±0.9^b^	7.11±0.84^b^	10.15±0.73^a^	<0.001
	TG (μmol/g)	14.75±1.72^a^	12.37±0.94^b^	13.8±1.64^ab^	14.58±1.8^a^	0.043
	LDL-C (μmol/g)	7.5±0.49^a^	6.52±0.39^b^	6.75±0.67^b^	7.04±0.33^ab^	0.027
	HDL-C (μmol/g)	2.66±0.35^c^	3.48±0.15^a^	3.28±0.2^ab^	3±0.28^b^	<0.001

a–c Means within a row lacking a common superscript differ (*P* < 0.05).

¹Data are presented as means ± SD (n = 6 geese per treatment). C = basal diet; N1 = basal diet + 0.06% NCG; N2 = basal diet + 0.08% NCG; N3 = basal diet + 0.12% NCG. NCG = N-carbamylglutamate.

²T-CHO = total cholesterol; TG = triglyceride; LDL-C = low-density lipoprotein cholesterol; HDL-C = high-density lipoprotein cholesterol.

### Serum and liver antioxidant biomarkers

Serum and liver antioxidant biomarkers are presented in Table 5. Serum SOD activity in the N1 group was significantly higher than that in the control group (*P* < 0.05), whereas no significant differences were observed between the N2, N3, and control groups. No significant differences were detected in serum GSH-Px activity among the four groups (*P* > 0.05). Serum MDA concentration in the N2 group was significantly lower than that in the control group (*P* < 0.05), whereas no significant differences were observed between the other NCG groups and the control group. Liver SOD activity in the N1 group was significantly higher than that in the control group (*P* < 0.05). Liver GSH-Px activity in the N1 group was significantly higher than that in the N3 group (*P* < 0.05), although no significant differences were observed between the control group and any of the NCG-supplemented groups. No significant differences were detected in liver MDA concentration among the four groups (*P* > 0.05).

**Table 5:** Effects of dietary N-carbamylglutamate supplementation on serum and liver antioxidant biomarkers of Huoyan geese¹.

**Tissue**	**Item²**	**C**	**N1**	**N2**	**N3**	**P-value**
Serum	SOD (U/mL)	53.08±2.42^b^	58.55±3.28^a^	57.21±3.11^ab^	55.55±4.76^ab^	0.048
	GSH-Px (U/mL)	680.95±36.73	696.59±30.67	668.96±27.79	680.07±20.02	0.671
	MDA (nmol/mL)	4.31±0.6^a^	3.74±0.55^ab^	3.64±0.35^b^	3.83±0.7^ab^	0.042
Liver	SOD (U/mg protein)	2.32±0.08^b^	2.48±0.19^a^	2.4±0.08^ab^	2.34±0.12^ab^	0.039
	GSH-Px (U/mg protein)	51.68±2.83^ab^	54.37±3.05^a^	52.09±3.19^ab^	51.33±2.25^b^	0.045
	MDA (nmol/mg protein)	1.07±0.14	1.05±0.07	1.05±0.04	1.08±0.11	0.892

a,b Means within a row lacking a common superscript differ (*P* < 0.05).

¹Data are presented as means ± SD (n = 6 geese per treatment). C = basal diet; N1 = basal diet + 0.06% NCG; N2 = basal diet + 0.08% NCG; N3 = basal diet + 0.12% NCG. NCG = N-carbamylglutamate.

²SOD = superoxide dismutase; GSH-Px = glutathione peroxidase; MDA = malondialdehyde.

### Liver transcriptomic profiles in response to NCG supplementation

To investigate the hepatic molecular response to NCG supplementation, liver tissue samples from the control and N1 groups were subjected to RNA-seq analysis, based on the superior egg quality outcomes observed in the N1 group. An average of approximately 44.07 million high-quality clean reads per sample was obtained, with Q30 values exceeding 96% (Supplementary Table S2). Differential expression analysis identified a total of 1,041 DEGs, of which 137 were upregulated and 904 were downregulated in the N1 group relative to the control (Fig. 1A). Hierarchical clustering analysis revealed that the overall expression patterns of DEGs in the NCG-supplemented group were clearly distinct from those in the control group (Fig. 1B).Principal component analysis separated the four control and four NCG libraries along PC1, which accounted for 36.56% of the variance of the dataset, with no overlap between the two groups (Fig. 1C). Correlation coefficients among the eight libraries were 0.948–0.978 within groups and 0.929–0.972 between groups (Fig. S1).

**Fig. 1:**
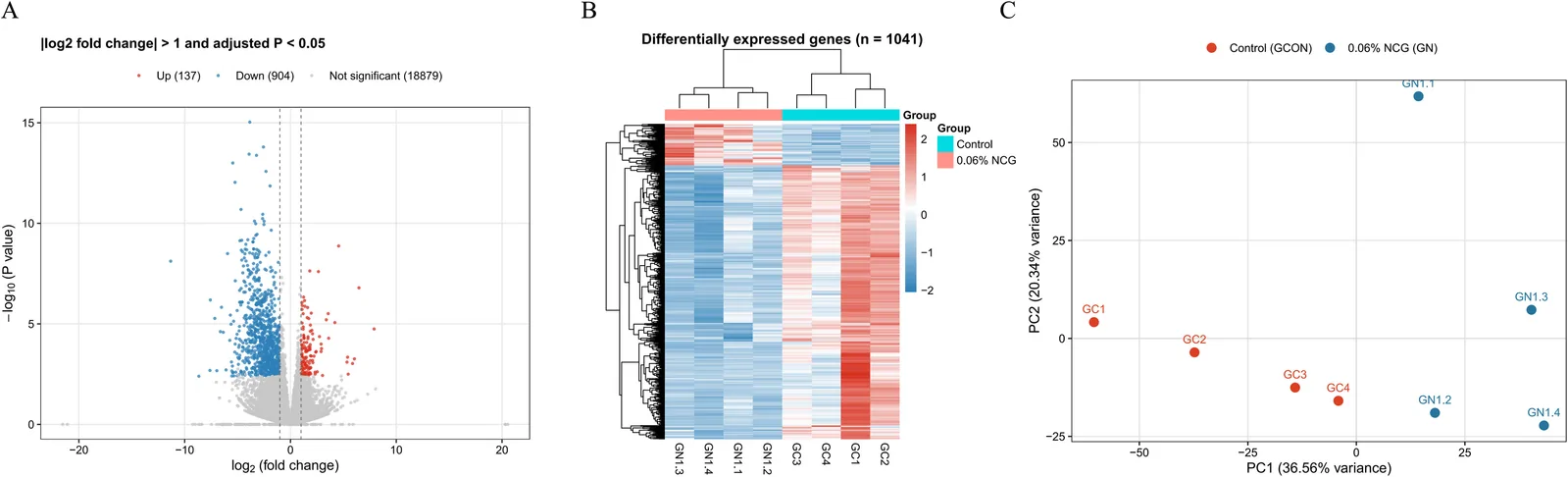
Liver transcriptome profile of the control and N1 groups. (A) Volcano plot of differentially expressed genes. (B) Hierarchical clustering heatmap of the differentially expressed genes. (C) Principal component analysis of the eight liver libraries.

### Functional enrichment analysis of DEGs

To explore the biological functions of the DEGs, GO and KEGG enrichment analyses were conducted. GO enrichment analysis indicated that the DEGs were predominantly associated with ribosome biogenesis and translation, involving genes such as *RPS18, RPS28, RPS27, RPL35A*, and *RPL36*. Additionally, GO terms related to the mitochondrial respiratory chain complex, the mitochondrial inner membrane and mitochondrial protein-containing complexes were among the most enriched terms (Fig. 2A, Table S4).

**Fig. 2:**
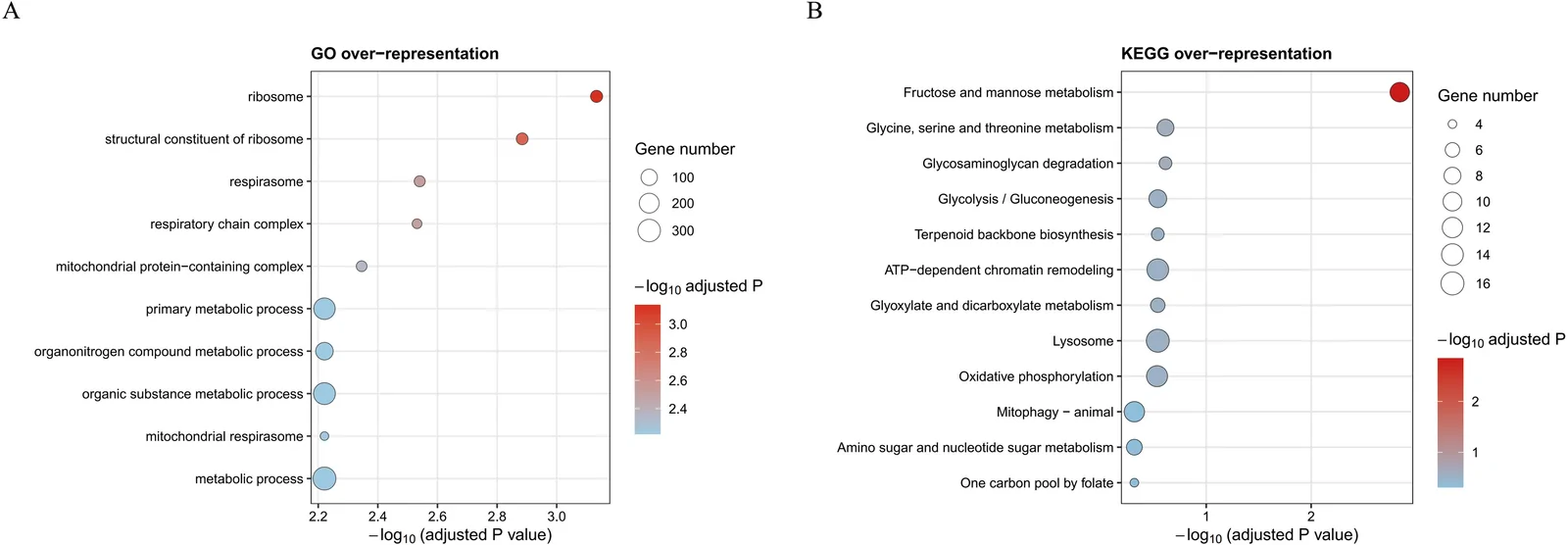
Gene Ontology (A) and KEGG (B) over-representation analysis of the differentially expressed genes.

KEGG over-representation analysis showed that the differentially expressed genes were nominally enriched in twelve pathways; after correction for multiple testing, fructose and mannose metabolism was the only pathway that remained significant (adjusted P = 0.001). The twelve most enriched pathways are shown in Fig. 2B and the complete results are given in Table S5.

### Gene set enrichment analysis of liver transcriptomic profiles

To identify coordinated pathway-level alterations across the entire transcriptome, GSEA was performed (Fig. 3). Gene sets with the most negative normalised enrichment scores in the N1 group included ribosome, glycerolipid metabolism, fructose and mannose metabolism, glycolysis/gluconeogenesis, carbon metabolism and galactose metabolism, whereas gene sets with positive scores included Polycomb repressive complex, progesterone-mediated oocyte maturation, cytokine-cytokine receptor interaction, ECM-receptor interaction and GnRH signalling (Fig. 3). Apart from ribosome (normalised enrichment score = −1.76, adjusted P = 0.031), no gene set reached an adjusted P < 0.05, so these coordinated changes are described as trends. These results indicate that NCG supplementation was accompanied by a coordinated suppression of ribosomal and carbohydrate metabolic gene sets rather than by the activation of a single pathway.

**Fig. 3:**
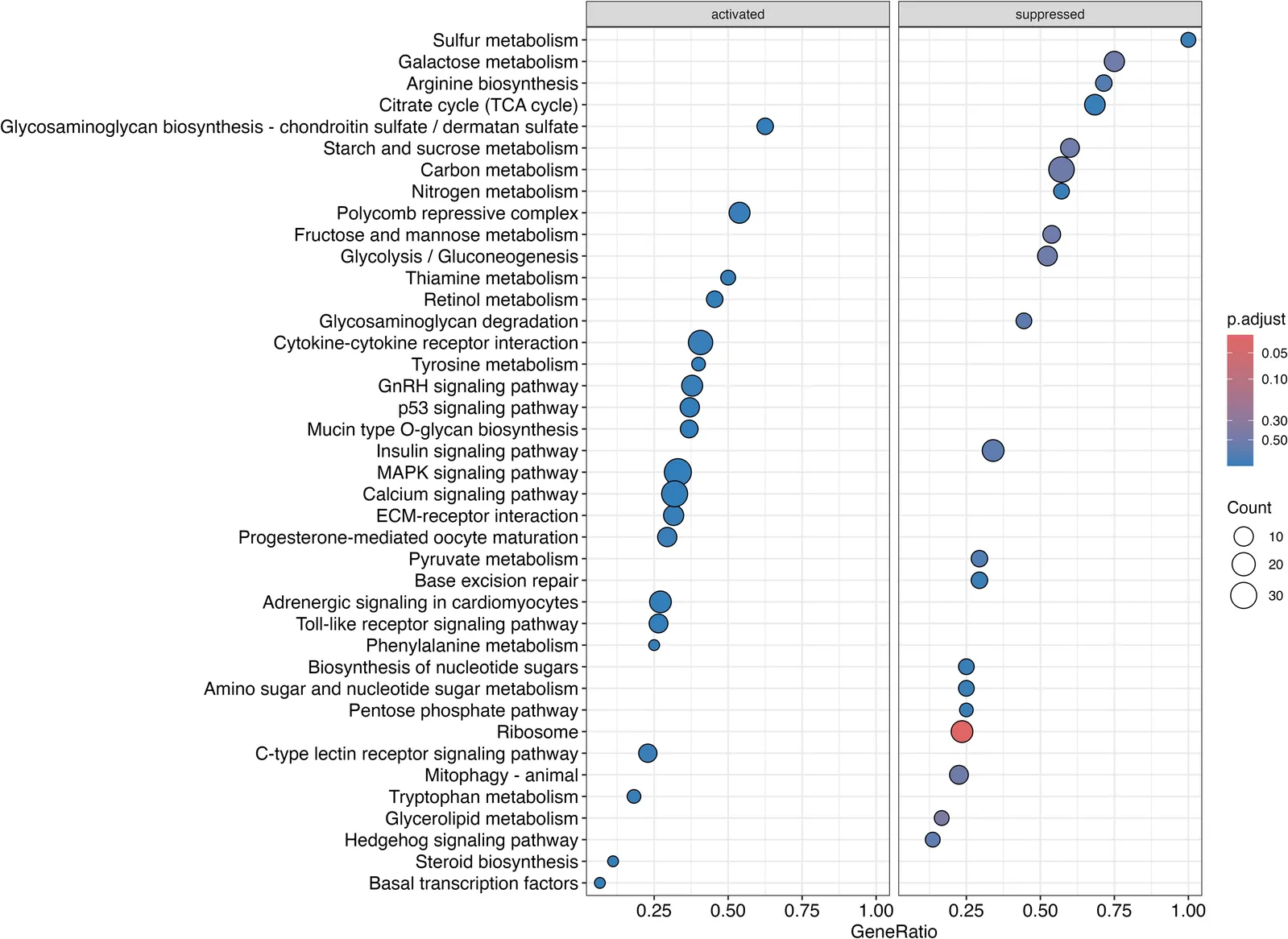
Gene set enrichment analysis of the liver transcriptome. Gene sets with activated and suppressed enrichment are shown in separate panels; dot size represents the number of genes in the gene set and colour represents the adjusted P value.

### qRT-PCR validation of RNA-seq results

To validate the reliability of the RNA-seq data, fourteen DEGs were selected for qRT-PCR analysis: *NDUFV1, TKFC, KEAP1, GSS, NOS3, COX5B, SDHC, RPS18, PRDX6, APOA2, EIF4B, CREB3L3, CFAP69*, and *SLC6A9* (Fig. 4). The qRT-PCR results showed that *NDUFV1, TKFC, KEAP1, GSS, NOS3, COX5B, SDHC, RPS18, PRDX6, APOA2, EIF4B, CREB3L3*, and *SLC6A9* were downregulated in the N1 group, whereas *CFAP69* was upregulated. The fold changes determined by qRT-PCR were consistent in direction with those obtained by RNA-seq for all fourteen genes, supporting the reliability of the transcriptomic findings.

**Fig. 4:**
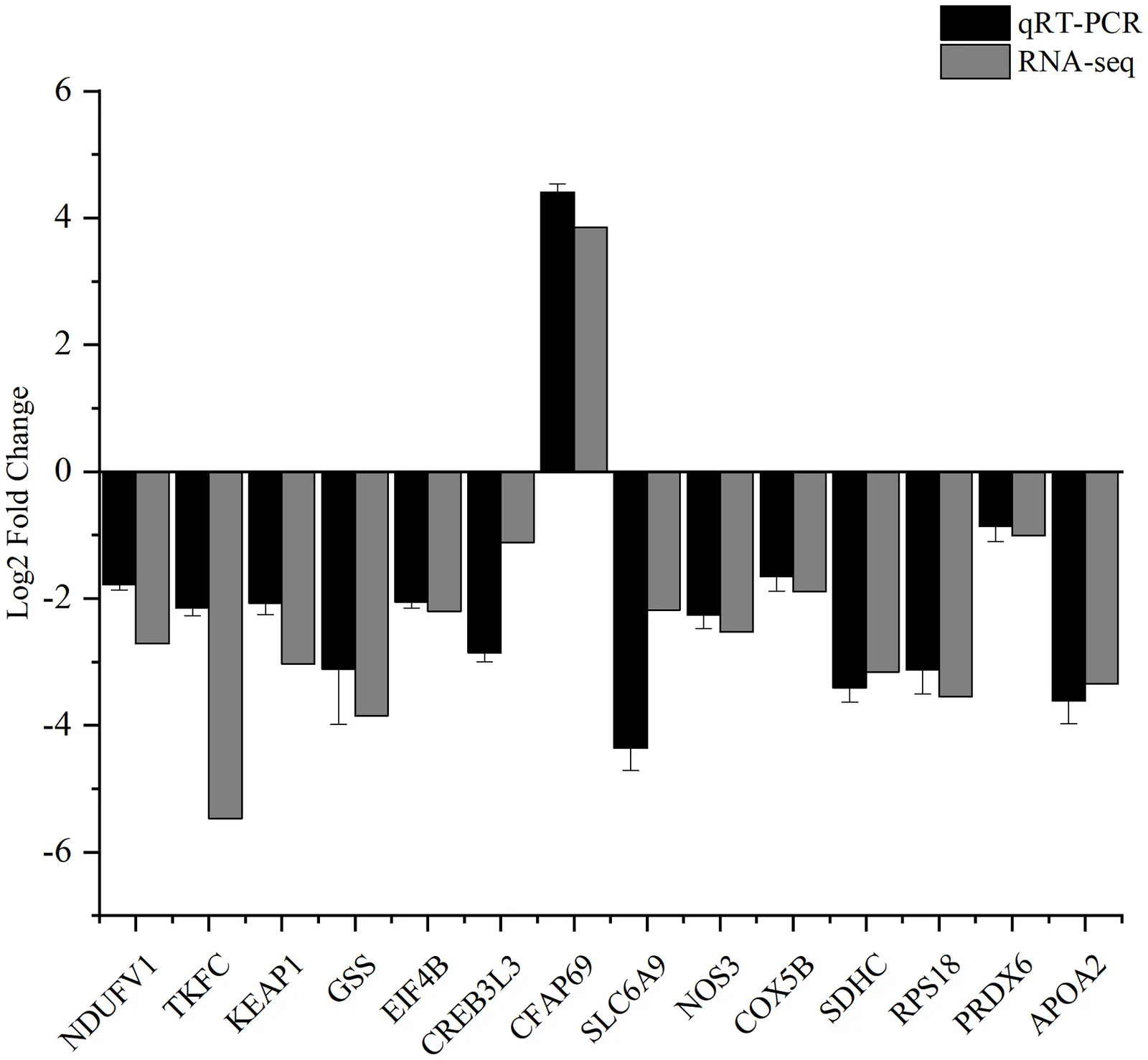
Validation of the RNA-seq results by qRT-PCR.

## Discussion

### Effects of NCG on laying performance

In the present study, the N1 group had a significantly higher laying rate and average egg weight than the control group, consistent with several previous reports in poultry. Zhang et al. (2021) reported that dietary NCG supplementation at 0.08%–0.16% significantly improved laying performance during peak production in Dagu chickens, with the 0.12% group producing optimal results. Hong et al. (2025) observed that supplementation with 1.0 g/kg NCG significantly increased laying rate and yolk TG and T-CHO contents in late-laying Huainan partridge chickens. Notably, the response to NCG in the present study was not monotonic: the N1 group showed the most pronounced improvements, whereas the laying rate in the N3 group did not differ significantly from the control. This pattern may be attributable to negative feedback regulation associated with the accumulation of arginine and its metabolites (e.g., ammonia, polyamines) in vivo; excessively high NCG doses may over-activate amino acid metabolic pathways, thereby disrupting systemic amino acid homeostasis.

### Effects of NCG on egg quality

NCG functions as an activator of endogenous arginine synthesis, promoting the conversion of glutamine or proline to citrulline via activation of P5CS and CPS-I, and consequently enhancing endogenous arginine production (Frank et al., 2007; Huang et al., 2019). Arginine is subsequently catalyzed by NOS to produce NO, a multifunctional signaling molecule that can dilate blood vessels, improve tissue blood flow, and enhance local microcirculation, thereby optimizing nutrient supply to the ovary and oviduct and promoting follicular development, ovulation, and albumen protein synthesis (Nguyen et al., 2023). In addition, arginine can regulate protein synthesis through the mTOR signaling pathway (Wang et al., 2018; Wu et al., 2009), which may contribute to the synthesis of egg albumen proteins and yolk lipoproteins and is a likely mechanism underlying the increased average egg weight observed in the present study.

Regarding egg quality, NCG supplementation significantly increased eggshell strength, eggshell thickness, and eggshell weight, with the N1 group showing a 45.3% increase in eggshell strength relative to the control. Ma et al.(2023a) similarly reported that 0.08% NCG supplementation significantly improved eggshell thickness in laying hens, and that the expression of calcium transport-related genes (*RYR2, CACNB2, RAMP3*, and *STAC*) in the uterus was significantly modulated by NCG. Eggshell formation depends primarily on efficient calcium ion transport in the shell gland (uterus), where calcium ions are transferred from blood to uterine luminal fluid via transcellular and paracellular pathways, and subsequently deposited as calcium carbonate on the organic matrix (Nys et al., 2004). These findings raise the possibility that NCG increases uterine blood flow through the arginine-NO pathway and thereby improves the efficiency of calcium supply, and that arginine serves as a precursor for collagen synthesis, reinforcing the structural integrity of the collagen matrix in the shell gland and providing a more stable scaffold for calcium carbonate deposition (Wu et al., 2009). Uterine blood flow, NO and NOS activity were not measured in this study, so this interpretation remains to be tested.

### Effects of NCG on lipid metabolism

The N1 group had significantly lower serum and liver T-CHO, TG and LDL-C concentrations and a significantly higher HDL-C concentration than the control group, indicating a positive regulatory effect on systemic lipid metabolism. These findings are corroborated by results from other species. Liu et al.(2024) reported that N2 supplementation in laying hens increased yolk polyunsaturated fatty acid, ω-3, and ω-6 fatty acid contents, and liver transcriptomic analysis identified upregulation of *CPT1A* (carnitine palmitoyltransferase 1A, the rate-limiting enzyme for fatty acid β-oxidation) and downregulation of *FASN* (fatty acid synthase, the key enzyme for de novo fatty acid synthesis). *CPT1A* is the critical transporter for long-chain fatty acid entry into mitochondria for β-oxidation, and its upregulation promotes fatty acid catabolism, whereas *FASN* catalyzes the synthesis of palmitate from acetyl-CoA and malonyl-CoA—a central step in the fatty acid synthesis pathway—and its downregulation reduces endogenous fatty acid production (Schultz et al., 2025). Hepatic *APOA2*, a major apolipoprotein of HDL, was downregulated in both RNA-seq and qRT-PCR, indicating that NCG also modified hepatic lipoprotein metabolism; this is consistent with the higher serum HDL-C concentration and the greater yolk weight observed in the N1 group. An alternative explanation for the lower hepatic TG content is enhanced VLDL synthesis and secretion, which would transport hepatic lipid to the growing ovarian follicles for yolk deposition rather than diverting it to intrahepatic catabolism; this is consistent with the greater yolk weight in the N1 group. The present data cannot distinguish this from increased hepatic lipid catabolism, because plasma VLDL and TG concentrations, hepatic *APOB* and *MTTP* expression, VLDL turnover and the yolk fatty acid profile were not measured.

One proposed route by which NCG could affect lipid metabolism is the arginine-NO-AMPK axis. In this model, NCG promotes endogenous arginine synthesis, and arginine is subsequently catalyzed by NOS to produce NO; NO can activate AMPK, and activated AMPK phosphorylates downstream targets to inhibit acetyl-CoA carboxylase (ACC) activity, reducing malonyl-CoA production, relieving its inhibitory effect on CPT1A and facilitating fatty acid entry into mitochondria for β-oxidation (Hardie et al., 2012). AMPK can also suppress the expression of lipogenic genes such as *FASN* by inhibiting the transcriptional activity of *SREBP-1c*. Hepatic *NOS3* was downregulated in both RNA-seq and qRT-PCR (Fig. 4), and neither NO and NOS activity nor AMPK phosphorylation and SREBP-1c activity was measured here, so increased hepatic NO signalling through *NOS3* could not be confirmed and this route remains a hypothesis that requires direct testing. In the present study, GSEA revealed a downregulation trend in the oxidative phosphorylation pathway following NCG supplementation, which may reflect a fine-tuned regulation of energy metabolism: when NCG promotes fatty acid oxidation and provides more adequate energy substrates, the basal oxidative phosphorylation level of mitochondria may be downregulated within a certain range to avoid excessive thermogenesis and increased reactive oxygen species (ROS) production.

### Effects of NCG on antioxidant capacity

Oxidative stress represents a significant metabolic challenge for laying poultry. In the present study, serum and liver SOD activities were significantly increased and serum MDA content was significantly decreased in the N1 group, indicating that NCG effectively enhanced antioxidant defense and reduced lipid peroxidation damage. Among the antioxidant genes validated, *PRDX6* was downregulated in both RNA-seq and qRT-PCR, showing that the transcriptional response of individual antioxidant genes was not uniform even though serum and hepatic SOD activities increased. Hu et al.(2023) demonstrated in weaned piglets that NCG treatment increased serum GSH-Px levels and decreased MDA levels, while significantly upregulating the mRNA expression of antioxidant enzyme-related genes (*CAT, SOD1, Gpx4,GCLC, GCLM*) and *Nrf2* in the jejunal mucosa. Studies in intrauterine growth-retarded (IUGR) lambs have similarly shown that NCG supplementation significantly increased liver GSH-Px and SOD activities and GSH content, decreased protein carbonyl and MDA levels, and enhanced antioxidant capacity by upregulating the mRNA expression of *GSH-Px1, SOD2, CAT, HO-1, iNOS*, and *eNOS* (Zhang et al., 2020).

Two mechanisms have been proposed for the antioxidant effect of NCG. The first is the arginine-NO pathway, in which NO has been proposed to act directly as an antioxidant. The second involves the KEAP1-Nrf2-ARE axis: under normal conditions Nrf2 is sequestered in the cytoplasm by KEAP1, and when KEAP1 is downregulated or its conformation is altered, Nrf2 is released, translocates to the nucleus and initiates transcription of antioxidant genes such as *SOD, CAT, GSH-Px, HO-1* and *NQO1* (Hu et al., 2023; Zhang et al., 2020). In the present dataset the only evidence for this axis is the downregulation of *KEAP1* in the N1 group; NO and NOS activity, Nrf2 protein abundance, its nuclear translocation and ARE activity were not measured, so the involvement of the KEAP1-Nrf2-ARE axis in the observed increase in SOD activity remains to be tested.

### Liver transcriptomics reveals core pathways of NCG-mediated metabolic regulation

Suppression of oxidative phosphorylation reflects adaptive changes in energy metabolism. GSEA revealed negative normalised enrichment scores for oxidative phosphorylation (OXPHOS), the citrate cycle, ribosome and PPAR signalling following NCG supplementation, ribosome reached an adjusted P < 0.05 (adjusted P = 0.031), whereas the other gene sets did not. Conventionally, promotion of oxidative phosphorylation implies enhanced energy metabolism; however, our results indicate that NCG suppressed the gene expression levels of OXPHOS and TCA cycle components. This is consistent with the downregulated expression of *COX5B* and *SDHC*, both of which were confirmed by qRT-PCR. When interpreted in conjunction with the significant decreases in T-CHO and TG and the significant increase in HDL-C observed in lipid metabolism biomarkers, this phenomenon likely reflects an adaptive metabolic shift under the influence of NCG. Increased fatty acid oxidation is one possible contributor to the lower hepatic TG content, but *CPT1A* was not differentially expressed in this dataset, the fatty acid degradation gene set was negatively enriched, and fatty acid oxidation flux was not measured (Murphy, 2009).

The PPAR signaling pathway is one of the core pathways governing lipid metabolism; *PPARα* activation upregulates the gene expression of fatty acid oxidation-related enzyme systems (Chen et al., 2021). NCG may activate AMPK and PPARα through the arginine-NO pathway; this remains a hypothesis, as neither activity was measured and the PPAR signalling and fatty acid degradation gene sets were negatively enriched. The downregulation of the ribosome pathway suggests that NCG may, to some extent, suppress the quantity rather than the quality of protein synthesis—i.e., reducing non-essential structural protein synthesis to redirect amino acid resources toward the synthesis of more functionally significant antioxidant and metabolic enzymes. This may explain why laying performance improved in parallel with the absence of significant changes in albumen height in the present study.

Enhanced glycolysis directs energy substrates toward glucose utilization. In marked contrast to the downregulation of energy metabolism pathways, GSEA analysis revealed that the upregulated gene set was enriched in fructose and mannose metabolism and glycolysis/gluconeogenesis. This indicates that NCG enhanced carbohydrate metabolic pathway activity while suppressing mitochondrial OXPHOS, potentially reflecting an adjustment in energy source and utilization strategy: the glycolytic pathway can rapidly generate ATP and channel carbon skeletons into intermediate metabolites, providing precursors for triglyceride synthesis, amino acid transamination, and nucleotide production (Stincone et al., 2015). In laying poultry, metabolic activity is intense during the peak laying period, and lipid metabolism is particularly active; the synthesis and transport of very-low-density lipoprotein (VLDL) is critical for yolk formation. This process involves large quantities of fatty acids and triglycerides in the liver, and NADPH—an essential reducing molecule for fatty acid synthesis—is necessarily supplied by the coordinated activity of the pentose phosphate pathway and carbohydrate metabolism (Hermier, 1997). The significant enrichment of the pentose phosphate pathway in the KEGG enrichment analysis in the present study supports this interpretation: the pentose phosphate pathway not only provides NADPH for lipid synthesis and antioxidant defense but also generates ribose-5-phosphate for nucleotide synthesis, meeting the substrate demands of follicular development and cell proliferation.

Glutathione metabolism and mitophagy jointly maintain cellular homeostasis. In the present dataset *GSS*, which encodes a rate-limiting enzyme of glutathione synthesis, was significantly downregulated in the N1 group and this was confirmed by qRT-PCR. This result shows that a pathway-level enrichment does not imply coordinated upregulation of every gene in the pathway, and it indicates that the increase in SOD activity was not accompanied by transcriptional activation of glutathione synthesis. Glutathione content and GSS activity were not measured, so compensatory post-transcriptional regulation cannot be excluded. Glutathione (GSH) is the most important non-enzymatic antioxidant in cells, and GSH-Px uses GSH as a substrate to eliminate hydrogen peroxide and organic peroxides (Brigelius-Flohé and Maiorino, 2013). The trend toward increased liver GSH-Px activity in the N1 group is not explained by transcriptional regulation of this pathway in the present dataset, because *GSS* was downregulated; post-transcriptional regulation of GSH-Px may be involved. The *KEAP1* gene was downregulated in the N1 group; the protein encoded by *KEAP1* is a negative regulator of Nrf2, and reduced *KEAP1* abundance may permit greater Nrf2 release and nuclear translocation, thereby initiating the transcription of downstream antioxidant genes. Nrf2 target genes include not only classical antioxidant enzymes such as *SOD, CAT*, and *GSH-Px* but also glutathione synthesis enzymes such as *GCLC* and *GCLM* (Schaupp et al., 2022), providing a transcriptional basis for the antioxidant effects of NCG.

The mitophagy gene set is another interesting aspect of the present dataset. Twelve mitophagy genes were over-represented among the differentially expressed genes (nominal *P* = 0.038; adjusted *P* = 0.48), whereas gene set enrichment analysis, which evaluates coordinated changes across the complete ranked list, showed a shift of the mitophagy gene set towards down-regulation (normalised enrichment score = −1.44; adjusted *P* = 0.51). Because over-representation analysis and gene set enrichment analysis answer different questions, the two results are not necessarily contradictory: a pathway may contain more differentially expressed genes than expected by chance while the gene set as a whole moves in the opposite direction, so the present data do not establish the direction of any functional change in mitophagy. Mitochondria are the principal source of intracellular ROS, and the selective clearance of damaged mitochondria contributes to mitochondrial quality control (Sies and Jones, 2020); it is therefore plausible, but not demonstrated, that NCG influences mitochondrial turnover. Transcript abundance cannot be used as a measure of autophagic flux, so whether mitophagy is altered by NCG remains a hypothesis that would require direct testing, for example measurement of the LC3B-II/LC3B-I ratio or p62/SQSTM1 abundance.

In conclusion, dietary NCG supplementation improved laying performance, egg quality, lipid metabolism, and antioxidant capacity in Huoyan geese, with 0.06% NCG identified as the recommended supplementation level under the present experimental conditions. Liver transcriptomic analysis revealed molecular changes associated with lipid metabolism, energy metabolism, antioxidant defense, and mitochondrial function. These findings provide a theoretical foundation for the application of NCG in goose production and offer insights for future optimization of poultry nutritional strategies to improve laying performance and metabolic health.

## Funding

This research was funded by the Liaoning Provincial Science and Technology Plan Joint Project Nos. 2025-MSLH-254, 2024-MSLH-144 and 2023-MSLH-037; Liaoning Provincial Science and Technology Special Commissioner Action Special Plan No. 2025JH5/10400041; Commissioned Special Project for the Construction of the Liaoning Innovation Team of the National Modern Agricultural Industrial Technology System (Broiler Industry), 2025.

## Data availability statement

The data presented in the study is deposited in the https://www.ncbi.nlm.nih.gov/ repository, accession number: PRJNA1478529.

## Ethics approval and consent to participate

All experimental procedures involving animals were approved by the Institutional Animal Care and Use Committee of Jinzhou Medical University (approval code: JZMULL2026131).

## Declaration of generative AI

No AI tools were used in this article.

## Author contributions

All authors meet the four authorship criteria. Each author reviewed and explicitly approved the exact final version of the manuscript and agrees to be personally and publicly accountable for all aspects of the work, ensuring that any questions regarding the accuracy or integrity of the research are thoroughly investigated and resolved. The specific contributions are as follows: **Xiaobing Peng** designed the animal experiment, conducted the animal trial and sample collection, analyzed transcriptomic and biochemical data, and drafted the manuscript. **Xuan Zhao** conducted the animal trial, sample collection and laboratory assays, and critically revised the manuscript. **Yizeng Lin** contributed to the experimental design and interpretation of production and egg-quality data, and critically revised the manuscript. **Meng Sun** performed laboratory assays, prepared figures and tables, and critically revised the manuscript. **Di Han** contributed to the collection and interpretation of production data, and critically revised the manuscript. **Changjiang Li** contributed to the collection and interpretation of production data, and critically revised the manuscript. **Ying Wang** performed library preparation and transcriptome data analysis, and critically revised the manuscript. **Chunchi Yan** contributed to the design and coordination of the animal trial and laboratory work, aided in data interpretation, and critically revised the manuscript. **Wei Ma** conceived the study, supervised the project, and critically revised the manuscript. **Chunqiang Wan**g conceived and designed the study, obtained funding, supervised the project, and critically revised the manuscript.

## Disclosures

The authors declare that they have no known competing financialinterests or personal relationships that could have appeared to influencethe work reported in this paper.

## Footnotes

Supplementary material associated with this article can be found, in the online version, at doi:10.1016/j.psj.2026.107981.
