# Supplementary material for: Dietary N-carbamylglutamate supplementation improves laying performance, lipid metabolism, and antioxidant capacity in Huoyan geese: insights from liver transcriptomics
Source: Poult Sci. 2026 Oct 17;105(12):107981. doi: 10.1016/j.psj.2026.107981 (PMC13721930; doi:10.1016/j.psj.2026.107981)
Supplement: Supplementary file 1 [file mmc2.docx]

Table S1. Sequences of the primers used for RT-qPCR.

| Gene | Primer information (5′-3′) | Fragment length | Reference gene sequence |
| --- | --- | --- | --- |
| *NDUFV1* | F: GACCGCATCTTCACCAACCTCTAC | 100 | XM_066998854.1 |
|  | R: CCTTCAGCAGGATCTCCTTCGTCT |  |  |
| *TKFC* | F: CTTCTGTGGTGCTGGTGGTGAA | 107 | XM_066998849.1 |
|  | R: GATGCGGACTCCTCGTTTCTCC |  |  |
| *KEAP1* | F: GAGGTCTCGAAGCAGGAGGAGTT | 119 | XM_066986462.1 |
|  | R: CAGTTGATGCAGGCGTGGAACA |  |  |
| *GSS* | F: AGGAAGGTGAAGGTGCGAGGAG | 192 | XM_066978414.1 |
|  | R: GCCACGAAGATGCTCAGACACAG |  |  |
| *EIF4B* | F: CGTGACCGAGGAGTCCATCAAG | 108 | XM_066985662.1 |
|  | R: GCGTAGCCAAACCCTTTCAACC |  |  |
| *CREB3L3* | F: GTGCTGACGGAGGACGAGAAGA | 132 | XM_048077816.2 |
|  | R: CGCTGACTGCTTGTTCCTGATCTT |  |  |
| *CFAP69* | F: TGGAGGCAGGAGGAATTGGACC | 119 | XM_048077480.2 |
|  | R: CGTCGTATGATAGCAAGCGTGACA |  |  |
| *SLC6A9* | F: TGGCTCCTGCTGATGGACAACT | 186 | XM_048059156.2 |
|  | R: GATGATGGCGGGCGAGATGAAG |  |  |
| *NOS3* | F: AGCGAGATCGGCACCAGGAA | 143 | XM_066991041.1 |
|  | R: AGCACGGCGATGTTCACCTC | 143 | XM_066991041.1 |
| *COX5B* | F: AAGTGCTGGAGGCCATGAACA | 171 | XM_066983559.1 |
|  | R: TGTGCAGCCAGAACCAGATGA | 171 | XM_066983559.1 |
| *SDHC* | F: GCTAAGTTCGCCCTGGCTTTCC | 102 | XM_066986083.1 |
|  | R: CTGCACCTGGCTGAGCTTGAAG | 102 | XM_066986083.1 |
| *RPS18* | F: TTCGCCATCACCGCCATCAAG | 193 | XM_066986885.1 |
|  | R: CCTTCACGTCCTTCTGCCTGTTG | 193 | XM_066986885.1 |
| *PRDX6* | F: CATCCGCTTCCACGACTTCCTG | 199 | XM_067001406.1 |
|  | R: GGCGTTGATGTCCTTGCTCCA | 199 | XM_067001406.1 |
| *APOA2* | F: CCACTTCCAGACCTTCTCCGACT | 105 | XM_048055496.2 |
|  | R: TGGTTGGCCTGCTCCAGATAGG | 105 | XM_048055496.2 |
| *β-actin* | F: TGTATGCCAACACGGTGCTGTC | 206 | XM_066977989.1 |
|  | R: CTCCTGCTTGCTGATCCACATCTG | 206 | XM_066977989.1 |
