# Supplementary material for: Dietary N-carbamylglutamate supplementation improves laying performance, lipid metabolism, and antioxidant capacity in Huoyan geese: insights from liver transcriptomics
Source: Poult Sci. 2026 Oct 17;105(12):107981. doi: 10.1016/j.psj.2026.107981 (PMC13721930; doi:10.1016/j.psj.2026.107981)
Supplement: Supplementary file 2 [file mmc3.docx]

**Table S2. Summary of the draft reads of the eight cDNA libraries, determined by RNA-sequencing.**

| Sample | Raw reads | Raw bases(G) | Clean reads | Clean bases(G) | Q30(%) | GC content(%) |
| --- | --- | --- | --- | --- | --- | --- |
| GC1 | 46748558 | 7.01 | 46310076 | 6.92 | 96.76 | 48.22 |
| GC2 | 42204326 | 6.33 | 41912910 | 6.25 | 97.21 | 47.64 |
| GC3 | 46013254 | 6.9 | 45748360 | 6.84 | 97.51 | 46.68 |
| GC4 | 39623000 | 5.94 | 39379234 | 5.88 | 97.07 | 46.06 |
| GN1_1 | 45105956 | 6.77 | 44614508 | 6.66 | 96.91 | 46.17 |
| GN1_2 | 44164004 | 6.62 | 43913496 | 6.54 | 97.42 | 46.63 |
| GN1_3 | 44798334 | 6.72 | 44461080 | 6.63 | 97.01 | 44.99 |
| GN1_4 | 46456468 | 6.97 | 46191548 | 6.88 | 97.55 | 45.94 |

Abbreviations: GC1–GC4: samples from the control group (C); GN1_1–GN1_4: samples from the 0.06% NCG-supplemented group (N1).
