# Supplementary material for: Dietary N-carbamylglutamate supplementation improves laying performance, lipid metabolism, and antioxidant capacity in Huoyan geese: insights from liver transcriptomics
Source: Poult Sci. 2026 Oct 17;105(12):107981. doi: 10.1016/j.psj.2026.107981 (PMC13721930; doi:10.1016/j.psj.2026.107981)
Supplement: Supplementary file 3 [file mmc4.docx]

**Table S3. Composition of the 1% pelleted premix used in the experimental diets**

**(a) Ingredients of the premix (kg per tonne of premix)**

| **Ingredient** | **0.06% NCG diet** | **0.08% NCG diet** | **0.12% NCG diet** |
| --- | --- | --- | --- |
| Vitamin premix | 20 | 20 | 20 |
| Mineral premix | 100 | 100 | 100 |
| Carrier | 513 | 493 | 453 |
| Choline chloride | 67 | 67 | 67 |
| DL-methionine | 140 | 140 | 140 |
| L-lysine (70%) | 80 | 80 | 80 |
| Phytase (10,000 FYT) | 20 | 20 | 20 |
| NCG | 60 | 80 | 120 |
| Total | 1000 | 1000 | 1000 |

**(b) Nutrients supplied by the premix per kilogram of complete diet**

| **Nutrient** | **Amountperkgdiet** | **Nutrient** | **Amountperkgdiet** |
| --- | --- | --- | --- |
| Fe | 53 mg | Vitamin A | 1.25 IU |
| Cu | 14 mg | Vitamin D₃ | 0.43 mg |
| Zn | 110 mg | Vitamin E | 125 mg |
| Se | 0.35 mg | Vitamin K₃ | 7.6 mg |
| Mn | 120 mg | Vitamin B₁ | 6.9 mg |
| I | 2.5 mg | Vitamin B₂ | 6.3 mg |
|  |  | Vitamin B₆ | 6.9 mg |
|  |  | Nicotinic acid | 63 mg |
|  |  | Folic acid | 4.5 mg |
|  |  | Calcium D-pantothenate | 21 mg |
|  |  | Vitamin B₁₂ | 0.07 mg |
|  |  | Biotin | 0.5 mg |
|  |  | Choline chloride | 400 mg |

*Note: The premix was included at 1% of the complete diet. Because the premix contained 60, 80 or 120 g NCG per kg premix, the complete diets contained 0.06%, 0.08% and 0.12% NCG, respectively; the amount of carrier was adjusted (513, 493 and 453 g/kg premix) so that the four premixtures, and therefore the four diets, were identical in all other respects.*
